# The association of parity number with multimorbidity and polypharmacy among Iranian women in the Azarcohort: a cross-sectional study

**DOI:** 10.1186/s12905-023-02434-9

**Published:** 2023-06-02

**Authors:** Elnaz Faramarzi, Mohammd Hossein Somi, Helda Tutunchi, Hanieh Almaspour, Sarvin Sanaie, Sanaz Asemani

**Affiliations:** 1 Liver and Gastrointestinal Diseases Research Center, Tabriz University of Medical Sciences, Tabriz, Iran; 2 Endocrine Research Center, Tabriz University of Medical Sciences, Tabriz, Iran; 3 Student Research Committee, Tabriz University of Medical Sciences, Tabriz, Iran; 4 Research Center for Integrative Medicine in Aging, Aging Research Institute, Tabriz University of Medical Sciences, Tabriz, Iran

**Keywords:** Parity, Multimorbidity, Polypharmacy, Pregnancy

## Abstract

**Objective:**

We aimed to study the association of parity number with multimorbidity (MM) and polypharmacy among women in the Azar cohort population.

**Patients & methods:**

This cross-sectional investigation was based on data from the Azar Cohort Study. Information regarding demographics, personal habits, physical activity level, medical and reproductive history, and anthropometric measurements of 8,290 females (35–70 years) were evaluated. Ordinal logistic and logistic regression analyses were conducted to assess for associations of parity number with multimorbidity (MM), polypharmacy, chronic disease, and abdominal obesity.

**Results:**

More educated participants and people in the fifth quintile of the Wealth Score Index were less likely to have a higher parity number. With increasing parity numbers, the prevalence of MM, polypharmacy, hypertension, cardiovascular disease, fatty liver disease, stroke, rheumatoid diseases, chronic obstructive pulmonary disease, and cancers tended to rise. Moreover, we found that increasing parity numbers (especially when ≥ 5) enhanced the odds of abdominal obesity, waist-to-hip ratio ≥ 0.85, and waist-to-height ratio ≥ 0.5; these significant associations were more obvious in parity numbers ≥ 9 and WHtR ≥ 0.5.

**Conclusion:**

The parity number is associated with MM and polypharmacy in Iranian women enrolled in the Azar Cohort Study. Further studies exploring the pathways (biological, social, and environmental) underlying these relationships will provide clues for preventing morbidity and premature mortality among susceptible andhighly parous women.

## Introduction

Pregnancy considerably affects a woman's physiological and behavioral status, leading to significant metabolic changes such as weight gain, dyslipidemia, increased blood glucose, and insulin resistance. Although some changes benefit both the mother and fetus, the pregnancy-related inflammatory state can, in the long term, lead to adverse health outcomes and promote several chronic diseases such as obesity, hypertension, cardiovascular diseases (CVDs), and diabetes [1, 2].

Multimorbidity (MM), defined as the coexistence of ≥ 2 chronic health conditions or diseases in an individual, is associated with poor outcomes and high healthcare costs [3]. Common risk factors include aging, obesity, an unhealthy lifestyle, and low socioeconomic status [4]. Polypharmacy, often defined as using five or more medications, is common among the older population (where multimorbidity is also more prevalent) and increases the risk of adverse medical outcomes [5]. With the increasing prevalence of concurrent morbidities in pregnancy, polypharmacy has risen in pregnant women [6]. To our knowledge, the association of parity with MM and polypharmacy is yet to be investigated; however, some studies have reported a significant association between parity and cardiometabolic disease. Recently, Moazzeni et al. [7] reported that Iranian women with ≥ 3 live births/parities had a higher risk of hypertension development than those with two live births or parities. In another study among rural Bangladeshi women, higher gravidity and parity were associated with an increased prevalence of metabolic syndrome (MetS), even after adjusting for potential confounders [8]. Moreover, in a large, pan-European cohort study, parous women had a higher risk of coronary heart disease than nulliparous women, with the highest risk observed among women with five or more children [9]. Nonetheless, evidence regarding the association of parity with chronic diseases and related complications is inconsistent. Although higher parity has been associated with an increased risk of chronic diseases such as type 2 diabetes mellitus (T2DM) [10–12], hypertension [7, 13], CVDs [7, 9], and MetS [2] in most previous studies, some studies indicate otherwise [14–17]. For instance, a prospective study found no significant association between the number of births and the risk of coronary heart disease [14].In another prospective study, Jacobsen et al. [15] found no consistent relationships between parity number and total mortality or mortality from ischemic heart disease or stroke. Furthermore, higher parity was not associated with a higher risk of MetSin Chinese normal-weight postmenopausal women [17]**.**

Although a significant positive association has also been reported between parity and obesity in several studies, controversial results have been found depending on the type of obesity (generalized or abdominal obesity). A cross-sectional survey of Chilean women indicated that parity modestly influenced the body mass index (BMI) but was not related to waist circumference (WC), waist-to-hip ratio (WHR), or waist-to-height ratio (WHtR) after controlling for confounders [18]. Other studies showed that abdominal obesity, but not BMI, was significantly associated with increased parities [19, 20]. However, Li et al. [21], found that higher parity was significantly associated with the risk of both general and abdominal obesity in middle-aged and older Chinese women.

To the best of our knowledge, no previous research has been conducted on the association of the number of pregnancies with MM and polypharmacy. Moreover, patterns of association may differ among various cultures, ethnic groups, and populations with different levels of development. Hence, we carried out this study to examine whether parity number is associated with MM and polypharmacy among women in the Iranian Azar population.

## Patients & methods

This cross-sectional investigation was based on baseline data collected from the Azar Cohort Study [22]. This ongoing cohort examines non-communicable diseases (NCDs) in the Shabestar Country of East Azarbaijan, Iran, as part of the Prospective Epidemiological Research Studies in Iran (PERSIAN) cohort [23]. This study has been performed in accordance with the Declaration of Helsinki, and ethical clearance was provided by the Ethics Committee of TabrizUniversity of Medical Sciences (IR.TBZMED.REC.1400.857). At the time of enrollment, written informed consent to participate in the study was obtained from participants (or their legal guardians in the case of illiterate participants). Enrollment phases commenced in 2014 andended in 2017. In the enrolment phase, 15,006 subjects were recruited. Living in the county for at least nine months was an inclusion criterion in the Azar Cohort Study. In addition, subjects who were not able to articulate their own opinions due to severe physical or psychiatric disorders were excluded from the enrollment phase of the Azar Cohort Study. We retrieved the baseline data pertaining to 8,290 female Shabestar County residents aged 35 to 70. Then, females who had never experienced menstruation were excluded from this study. Ultimately, 8,286 females were included in our study. Further details concerning the Azar Cohort Study are published elsewhere [23].

### Demographic characteristics

Data were collected using a questionnaire. Demographic data (age, sex, marital status, education, etc.) were assessed along with lifestyle habits such as smoking. Smokers were considered as those who constantly smoked a minimum of one cigarette per day for over half a year; non-smokers had no history of smoking or had quit; and users of other tobacco products were those who smoked hookah, water pipe, pipe, or chewed nass.

The Wealth Score Index (WSI) was determined via Multiple Correspondence Analysis (MCA) to delineate the participants' socioeconomic status. This score was based on assets (e.g., dishwashers, vehicles, and flat-screen TVs), household conditions (e.g., the number of rooms, type of ownership), and education level. We divided the subjects into five SES quintiles, with the first being the lowest and the fifth being the highest.

Daily activity was investigated according to metabolic equivalent tasks (METs) with a questionnaire. METs represent the energy consumed by a person considering their weight. For example, one MET equates to the oxygen volume utilized by an individual at rest per kg per min (3.5 ml), while 4 METsequal 16 ml/kg/min.

### Reproductive history

A comprehensive reproductive history (including menarche, parity, pregnancy number, breastfeeding duration, menopause, oral contraceptive pills (OCP), and hormone replacement therapy) was asked of each participant. The parity number was defined as the number of pregnancies (successful or unsuccessful)reaching beyond 20 weeks. A miscarriage was defined as losing a pregnancy before the 20^th^ week of gestation. We categorized the women according to their parity number into groups 1 (no parity history), 2 (1–2parities), 3 (3–4 parities), 4 (5–6 parities), 5 (7–8parities), and 6 (≥ 9 parities).

### Multimorbidity

Multimorbidity (MM) was defined as the simultaneous occurrence of at least two chronic diseases, including obesity, diabetes, hypertension, cardiovascular diseases (CVDs), stroke, chronic obstructive pulmonary diseases (COPD), cancer (gastrointestinal, breast, ovarian, skin, bladder, lung, head and neck, or hematopoietic), depression, fatty liver disease, or rheumatoid diseases. A history of a doctor telling the subject that they had each disease was asked in the questionnaire.

### Polypharmacy

Polypharmacy is usually defined as the daily use of at least five medications for at least three months [5]. Our questionnaire asked about all prescribed, over-the-counter, and dietary or herbal drugs. Also, contraceptives or hormone replacement therapy were counted as drugs in the variable polypharmacy.

### Anthropometric measurements

After measuring the height and weight of each participant, the BMI was calculated by dividing the weight (kg) by the height (m) squared. The World Health Organization (WHO) classification of BMI was used, where values below 18.5 kg/m^2^ indicate an underweight status, values of 18.5–24.9 kg/m^2^ are considered normal, and being overweight or obese is signaled by values of 25–29.9 and ≥ 30 kg/m^2^, respectively. We followed the standards set by the National Institutes of Health in measuring waist circumference (WC) and hip circumference, then calculated the waist-to-hip ratio (WHR) and waist-to-height ratio (WHtR). Indicessignaling abdominal obesity among our female subjects included WC ≥ 88 cm [24] and WHR ≥ 0.85 cm [25], or WHtR ≥ 0.5 [26].

### Statistical analysis

Four of the 8,290 female subjects were excluded because they had never experienced menstruation. Hence, statistical analysis was done using data on 8,286 women, classified according to the parity number. Descriptive statistics are reported as frequency and percentage or mean ± standard deviation (SD)as appropriate. To assess for differences in the study variables across the groups, the analysis of variance (ANOVA), chi-squared, and Kruskal–Wallis tests were recruited where appropriate. Ordinal regression was used to assess for evidence of the association of parity number with MM and polypharmacy, and logistic regression was used to check its relationship with chronic disease and abdominal obesity, with the odds ratios (ORs) and corresponding 95% confidence intervals (CIs) being reported. Two models were used: Model 1 – unadjusted; Model 2 – adjusted for age, education level, WSI, residential area, METs, and marital status. *P* < 0.05 indicated statistical significance, and SPSS v. 20 (SPSS Inc., Chicago, IL, USA) was used for all analyses.

## Results

Our analysis included 8,286 women with a mean age of 49.24 ± 9.26 years. Women were grouped according to the parity number: nulliparous women (486; 5.9%); women with 1–2 parities (2709; 32.71%), 3–4 parities (2985; 36.0%), 5–6 parities (1,158; 14%), 7–8 parities (554; 6.7%), and ≥ 9 parities (394; 4.8%). Baseline characteristics are presented in Table 1 according to the parity number. The more educated and wealthy (fifth quintile of WSI) participants were less likely to have a higher parity number. Among the categorical variables, increased parity was associated with abdominal obesity (WC ≥ 88 cm), generalized obesity, and elevated WHR and WHtR.Age, WC, BMI, WHR, and WHtRin women without any history of parity or 1–2 parities were significantly lower than in the other groups(*P* < 0.05). Generally, with a rise in parity number, the prevalence of MM, polypharmacy, hypertension, CVD, fatty liver disease, stroke, rheumatoid diseases, COPD, and cancer tended to increase (Table 2).

**Table 1:** General characteristics of participants stratified by number of parity

	**0 (*n* = 486)**	**1–2(*n* = 2709)**	**3–4 (*n* = 2985)**	**5–6 (*n* = 1158)**	**7–8 (*n* = 554)**	** ≥ 9 (*n* = 394)**	***P* value**
	N(%)	N(%)	N(%)	N(%)	N(%)	N(%)	
**Age classification**							< 0.001*
35–49	319(65.6)	2279(84.1)	1744(58.4)	156(13.5)	11(2.0)	1(0.3)	
50–59	121(24.9)	380(14)	1015(34)	621(53.6)	209(37.7)	61(15.5)	
60–70	46(9.5)	50(1.8)	226(7.6)	381(32.9)	334(60.3)	332(84.3)	
**Education level**							< 0.001*
Illiterate	63(13.0)	121(4.5)	470(15.8)	550(47.5)	398(72)	316(80.2)	
Primary school	218(44.9)	942(34.8)	1536(51.5)	487(42.1)	138(25)	70(17.8)	
Diploma	149(30.7)	1287(47.5)	897(30.1)	118(10.2)	17(3.1)	8(2.0)	
University	56(11.5)	357(13.2)	80(2.7)	2(0.2)	0(0.0)	0(0)	
**Marital Status**							< 0.001**
Married	241(49.6)	2532(93.5)	2765(92.6)	983(84.9)	445(80.3)	305(77.4)	
Unmarried	245(50.4)	177(6.5)	220(7.4)	175(15.1)	109(19.7)	89(22.6)	
**Quintiles of wealth index**							< 0.001*
1(poorest)	234(48.1)	570(21.0)	652(21.8)	416(35.9)	245(44.2)	163(41.4)	
2	103(21.2)	342(12.6)	484(16.2)	285(24.6)	152(27.4)	108(27.4)	
3	65(13.4)	514(19.0)	653(21.9)	223(19.3)	96(17.3)	78(19.8)	
4	43(8.8)	765(28.2)	704(23.6)	132(11.4)	28(5.1)	31(7.9)	
5 (richest)	41(8.4)	518(19.1)	492(16.5)	102(8.8)	33(6.0)	14(3.6)	
**Residential regions**							< 0.001**
Urban residents	312(64.2)	2080(76.8)	2093(70.1)	702(60.6)	311(56.1)	203(51.5)	
Rural residents	174(35.8)	629(23.2)	892(29.9)	456(39.4)	243(43.9)	191(48.5)	
**Physical activity level (METs**^**a**^**)**							< 0.001*
Low	211(43.4)	911(33.6)	923(31.1)	430(37.2)	277(50)	212(53.8)	
Moderate	182(37.4)	1265(46.7)	1392(46.7)	493(42.6)	193(34.8)	130(33.0)	
High	93(19.1)	533(19.7)	667(22.4)	234(20.2)	84(15.2)	52(13.2)	
**Current smoking status**							0.59*
No smoker	481 (99.0)	2690(99.3)	2961(99.2)	1154(99.7)	550(99.3)	390(99.0)	
Smoker	2(0.4)	11(0.4)	17(0.4)	2(0.2)	3(0.5)	4(1)	
Smokerother type of tobacco products	3(0.6)	8(0.3)	7(0.2)	2(0.2)	1(0.2)	0(0)	
Use of oral contraceptive	42(8.6)	2029(74.9)	2507(84.0)	923(81.2)	374(67.5)	244(61.9)	< 0.001**
Use of hormone therapy	54(11.1)	209(7.7)	163(5.5)	36(3.1)	11(2.0)	2(0.5)	< 0.001**
**WC ≥ 88**^**c**^	230(47.4)	1604(59.2)	2198(73.7)	931(80.4)	454(82.1)	314(79.7)	< 0.001**
**WHR ≥ 0.85**^**d**^	228(47.0)	1412(52.1)	1962(65.8)	934(80.7)	478(86.4)	350(88.8)	< 0.001**
**WHtR ≥ 0.5**^**e**^	377(77.7)	2384(88.0)	2820(94.5)	1120(96.7)	541(97.8)	383(97.2)	< 0.001**
**BMI (kg/m**^**2**^**) classification**^**b**^							< 0.001*
Underweight < 18.5	8(1.7)	8(0.3)	6(0.2)	5(0.4)	1(0.2)	1(0.3)	
Normal weight 18.5–24.9	131(27.6)	492(18.4)	333(11.3)	122(10.7))	58(10.6)	60(15.5)	
Overweight 25–29.9	182(38.3)	1099(41.2)	1066(36.1)	383(33.5)	200(36.5)	126(32.6)	
Obese ≥ 30	154(32.4)	1069(40.1)	1548(52.4)	634(55.4)	289(52.7)	200(51.7)	
	**mean ± SD**	**mean ± SD**	**mean ± SD**	**mean ± SD**	**mean ± SD**	**mean ± SD**	
Age (years)	46.81 ± 8.57	43.05 ± 6.33	48.31 ± 7.41	56.59 ± 6.54	61.04 ± 5.24	63.69 ± 4.07	< 0.001^***^
Height (cm)	155.71 ± 6.91	157.66 ± 5.94	156.37 ± 5.73	153.98 ± 5.69	152.56 ± 5.77	152.51 ± 5.61	< 0.001***
Weight (kg)	67.78 ± 13.51	72.64 ± 12.96	74.44 ± 12.56	73.13 ± 13.49	71.68 ± 13.01	70.97 ± 13.56	< 0.001^***^
Waist circumference	87.42 ± 12.35	90.83 ± 11.01	94.13 ± 10.59	96.55 ± 11.09	97.86 ± 11.37	97.47 ± 11.36	< 0.001^***^
Hip circumference	103.01 ± 9.79	106.32 ± 9.40	107.13 ± 9.22	106.12 ± 10.65	104.91 ± 10.30	104.20 ± 10.66	< 0.001^***^
BMI (kg/m^2^)^b^	27.91 ± 5.53	29.21 ± 5.02	30.41 ± 4.79	30.80 ± 5.22	30.71 ± 5.31	30.49 ± 5.43	< 0.001^***^
WHR^d^	0.84 ± 0.08	0.85 ± 0.07	0.87 ± 0.07	0.91 ± 0.07	0.93 ± 0.07	0.93 ± 0.07	< 0.001^***^
WHtR^e^	0.56 ± 0.08	0.57 ± 0.07	0.60 ± 0.06	0.62 ± 0.07	0.64 ± 0.07	0.63 ± 0.07	< 0.001^***^

^*^P kruskal Wallis tes

^**^P chi-square test

^***^P One Way ANOVA

^a^*MET* Metabolic Equivalent Of Task

^b^*BMI* body mass index

^c^*WC* waist circumference

^d^*WHR* waist to hip ratio

^e^*WHtR* waist to height ratio

**Table 2:** Prevalence of multimorbidty, polypharmacy, and chronic diseases based on number of parity

	**0 (*n* = 486)**	**1–2(*n* = 2709)**	**3–4 (*n* = 2985)**	**5–6 (*n* = 1158)**	**7–8 (*n* = 554)**	** ≥ 9 (*n* = 394)**	***P* value**
	N(%)	N(%)	N(%)	N(%)	N(%)	N(%)	
Number of chronic diseases							< 0.001*
**0**	225(46.3)	1071(39.5)	791(26.5)	199(17.2)	80(14.4)	57(14.5)	
**1**	150(30.9)	996(36.8)	1105(37.0)	352(30.4)	154(27.8)	93(23.7)	
**≥ 2**(multimorbidity)	111(22.8)	642(23.7)	1089(36.5)	607(52.4)	320(57.8)	242(61.7)	
**Polypharmacy**							< 0.001*
0	282(58.0)	1610(59.4)	1559(52.2)	435(37.6)	182(32.9)	125(31.7)	
1	83(17.1)	515(19.0)	593(19.9)	228(19.7)	92(16.6)	68(17.3)	
2	44(9.1)	279(10.3)	361(12.1)	160(13.8)	75(13.5)	50(12.7)	
3	29(6.0)	134(4.9)	155(5.2)	84(7.3)	41(7.4)	26(6.6)	
4	8(1.6)	60(2.2)	74(2.5)	47(4.1)	33(0.6)	24(6.1)	
≥ 5	40(8.2)	111(4.1)	2438(8.1)	204(17.6)	131(23.6)	101(25.6)	
Hypertension	82(16.9)	332(12.3)	678(22.7)	463(40)	274(49.5)	217(55.1)	< 0.001**
Diabetes	29(6.0)	180(6.6)	319(10.7)	260(22.5)	147(26.5)	118(29.9)	< 0.001**
CVD	13(2.7)	33(1.2)	104(3.5)	83(7.2)	60(10.8)	50(12.7)	< 0.001**
Fatty liver	16(3.3)	115(4.2)	174(5.8)	90(7.8)	37(6.7)	35(8.9)	< 0.001**
Stroke	1(0.2)	8(0.3)	16(0.5)	18(1.6)	9(1.6)	12(3.0)	< 0.001**
Depression	93(19.1)	597(22.0)	762(25.5)	316 (27.3)	160(28.9)	96(24.4)	< 0.001**
Rheumatoid Disease	15(3.1)	111(4.1)	135(4.5)	77(6.6)	28(5.1)	23(5.8)	0.006**
COPD	20(4.1)	74(2.7)	113(3.8)	67(5.8)	34(6.1)	39(9.9)	< 0.001**
Cancer	3(0.6)	13(0.5)	17(0.6)	11(0.9)	9(1.6)	7(1.8)	0.006**

^*^P kruskal Wallis test

^**^P chi-square test

Findings of regression for the association of parity number with MM, polypharmacy, and various chronic diseases are presented in Table 3. Compared to women without parity, women with parity numbers of 3–4 (OR: 2.21; 1.84–2.65), 5–6(OR:4.12; 3.36–5.04), 7–8 (OR:5.12;4.04–6.48), and ≥ 9 (OR:5.87;4.51–7.63)had significantly higher odds of MM. This association remained significant even after further adjustments for confounding factors. Women with 7–8(OR: 3.26;2.58–4.11)or ≥ 9(OR:3.47;2.70–4.47)parities had a significantlyhigher OR of polypharmacy than nulliparous women. Moreover, compared to those with no parity, women with ≥ 5 parities had a higher adjusted OR for polypharmacy (Table 3).

**Table 3:** The association between multimorbidity, ploypahrmacy, chronic diseases, and abdominal obesity with number of parity

	**Number of parity**										
	0	1–2	P	3–4	P	5–6	P	7–8	P	≥ 9	P
	N(%)	N(%)		N(%)		N(%)		N(%)		N(%)	
**Multimorbidity**											
Model^1^	Reference	1.22(1.01–1.46)	0.03	2.21(1.84–2.65)	< 0.001	4.12(3.36–5.04)	< 0.001	5.12(4.04–6.48)	< 0.001	5.87(4.51–7.63)	< 0.001
Model^2^	Reference	1.46(1.20–1.78)	0.001	1.90(1.56–2.31)	< 0.001	2.28(1.82–2.85)	< 0.001	2.19(1.68–2.85)	< 0.001	2.18(1.62–2.93)	< 0.001
**Poly pharmacy**											
Model^1^	Reference	0.88(0.73–1.06)	0.19	1.21(1.00–1.45)	0.04	2.36(1.92–2.89)	< 0.001	3.26(2.58–4.11)	< 0.001	3.47(2.70–4.47)	< 0.001
Model^2^	Reference	1.07(0.88–1.32)	0.46	1.09(0.89–1.34)	0.35	1.34(1.07–1.68)	0.01	1.38(1.07–1.79)	0.01	1.22(0.91–1.62)	0.17
**Hypertension**											
Model^1^	Reference	0.68(0.52–0.89)	0.005	1.44(1.12–1.86)	0.004	3.28(2.51–4.27)	< 0.001	4.82(3.60–6.44)	< 0.001	6.04(4.43–8.23)	< 0.001
Model^2^	Reference	1.18(0.88–1.58)	0.26	1.36(1.02–1.80)	0.03	1.37(1.02–1.85)	0.03	1.26(0.90–1.75)	0.16	1.20(0.84–1.72)	0.29
**Diabetes**											
Model^1^	Reference	1.12(0.74–1.68)	0.57	1.88(1.27–2.79)	0.002	4.56(3.05–6.80)	< 0.001	5.69(3.74–8.66)	< 0.001	6.73(4.37–10.38)	< 0.001
Model^2^	Reference	1.56(1.02–2.37)	0.03	1.74(1.15–2.61)	0.008	2.65(1.74–4.04)	< 0.001	2.54(1.62–3.99)	< 0.001	2.65(1.65–4.24)	< 0.001
**Stroke**											
Model^1^	Reference	1.43(0.17–11.51)	0.73	2.61(0.34–19.75)	0.35	7.65(1.01–57.52)	0.05	8.0(1.01–63.44)	0.04	15.23(1.97–117.68)	0.009
Model^2^	Reference	2.06(0.24–17.16)	0.50	2.11(0.27–16.29)	0.47	3.05(0.39–23.72)	0.28	2.09(0.24–17.57)	0.49	3.26(0.39–27.15)	0.27
**CVD**											
Model^1^	Reference	0.44(0.23–0.85)	0.01	1.31(0.73–2.35)	0.36	2.80(1.55–5.09)	0.001	4.41(2.39–8.15)	< 0.001	5.28(2.82–9.88)	< 0.001
Model^2^	Reference	0.78(0.39–1.53)	0.47	1.38(0.75–2.54)	0.29	1.49(0.79–2.81)	0.20	1.63(0.84–3.18)	0.14	1.62(0.81–3.22)	0.16
**Fatty liver**											
Model^1^	Reference	1.30(0.76–2.21)	0.33	1.81(1.08–3.06)	0.02	2.47(1.43–4.25)	0.001	2.10(1.15–3.82)	0.01	2.86(1.56–5.25)	0.001
Model^2^	Reference	1.45(0.83–2.52)	0.18	1.67(0.97–2.88)	0.06	1.88(1.05–3.34)	0.03	1.43(0.75–2.75)	0.27	1.84(0.94–3.60)	0.07
**Cancers**											
Model^1^	Reference	0.77(0.22–2.73)	0.69	0.92(0.26–3.15)	0.89	1.54(0.42–5.55)	0.50	2.65(0.71–9.87)	0.14	2.91(0.74–11.33)	0.12
Model^2^	Reference	0.93(0.25–3.48)	0.92	0.92(0.25–3.34)	0.90	1.01(0.25–4.02)	0.98	1.33(0.31–5.75)	0.69	1.24(0.27–5.73)	0.77
**Depression**											
Model^1^	Reference	1.19(0.93–1.52)	0.15	1.44(1.13–1.84)	0.003	1.58(1.22–2.05)	0.001	1.71(1.28–2.29)	< 0.001	1.36(0.98–1.8)	0.06
Model^2^	Reference	1.28(0.99–1.66)	0.05	1.47(1.14–1.91)	0.003	1.58(1.19–2.11)	0.002	1.67(1.20–2.32)	0.001	1.29(0.90–1.86)	0.16
**Rheumatoid Disease**											
Model^1^	Reference	1.34(0.77–2.32)	0.29	1.48(0.86–2.55)	0.15	2.23(1.27–3.93)	0.005	1.67(0.88–3.16)	0.11	1.94(1.00–3.78)	0.04
Model^2^	Reference	1.24(0.70–2.20)	0.44	1.22(0.69–2.14)	0.48	1.53(0.84–2.79)	0.16	1.05(0.53–2.11)	0.87	1.17(0.56–2.43)	0.66
**COPD**											
Model^1^	Reference	0.65(0.39–1.08)	0.09	0.91(0.56–1.49)	0.72	1.43(0.85–2.38)	0.16	1.52(0.86–2.68)	0.14	2.56(1.46–4.46)	0.001
Model^2^	Reference	0.64(0.37–1.09)	0.10	0.89(0.53–1.49)	0.66	1.24(0.71–2.18)	0.44	1.19(0.63–2.26)	0.58	1.93(1.01–3.68)	0.04
**Obesity**											
Model^1^	Reference	1.40(1.14–1.72)	0.001	2.32(1.89–2.84)	< 0.001	2.60(2.08–3.25)	< 0.001	2.35(1.82–3.03)	< 0.001	2.22(1.68–2.92)	< 0.001
Model^2^	Reference	1.35(1.08–1.68)	0.007	1.98(1.59–2.46)	< 0.001	2.15(1.69–2.75)	< 0.001	1.91(1.44–2.54)	< 0.001	1.82(1.33–2.48)	< 0.001
**Abdominal obesity (WC ≥ 88 cm)**											
Model^1^	Reference	1.61(1.32–1.95)	< 0.001	3.10(2.54–3.77)	< 0.001	4.54(3.61–5.72)	< 0.001	5.08(3.83–6.73)	< 0.001	4.35(3.21–5.89)	< 0.001
Model^2^	Reference	1.70(1.37–2.10)	< 0.001	2.52(2.03–3.13)	< 0.001	2.78(2.15–3.59)	< 0.001	2.67(1.94–3.66)	< 0.001	2.09(1.48–2.96)	< 0.001
**WHR ≥ 0.85**											
Model^1^	Reference	1.22(1.01–1.49)	0.03	2.16(1.78–2.62)	< 0.001	4.70(3.73–5.91)	< 0.001	7.18(5.31–9.71)	< 0.001	8.96(6.25–12.86)	< 0.001
Model^2^	Reference	1.56(1.26–1.95)	< 0.001	1.86(1.49–2.32)	< 0.001	2.20(1.69–2.85)	< 0.001	2.34(1.67–3.28)	< 0.001	2.40(1.61–3.58)	< 0.001
**WHtR ≥ 0.5**											
Model^1^	Reference	2.10(1.65–2.68)	< 0.001	4.92(3.77–6.42)	< 0.001	8.44(5.73–12.44)	< 0.001	12.91(7.01–23.78)	< 0.001	9.97(5.27–18.84)	< 0.001
Model^2^	Reference	1.97(1.47–2.65)	< 0.001	3.22(2.35–4.41)	< 0.001	3.76(2.40–5.86)	< 0.001	4.79(2.44–9.39)	< 0.001	3.25(1.60–6.64)	< 0.001

Model1 was unadjusted

Model2 was adjusted for age, education level, WSI (wealth score index), Residential regions, marital status,metabolic equivalents task (METs)

The OR of hypertension increased in women with 5–6, 7–8, and ≥ 9 parities relative to nulliparous women. Compared to nulliparous women, women with ≥ 9 parities had a 6.73[4.37–10.38] times higher OR of diabetes in Model 1; in Model 2, after further adjustments, the corresponding OR fell to 2.65[1.65–4.24], yet remained significant.

Interestingly, after adjusting for confounding factors, the adjusted OR of depression in women with ≥ 3 parities was significantly higher than in nulliparous women. Compared to nulliparous women, women with ≥ 9 parities had a 1.93 times higher OR of COPD after further adjustments (OR: 1.93, 95%CI 1.01–3.68).

We found that increasing parity numbers (especially ≥ 5) enhanced the adjusted OR of abdominal obesity, WHR ≥ 0.85, and WHtR ≥ 0.5. In other words, women with parity numbers ≥ 9 had the highest odds of WHR ≥ 0.85 (OR:2.40, 95% CI 1.61–3.58).

## Discussion

The present study found that with a rise in the parity number, the prevalence of MM and polypharmacy tended to increase. As far as we know, no previous research has been conducted on the association of the number of pregnancies with MM and polypharmacy. We postulated some assumptions. Firstly, increasing parity numbers may redistribute fat and cause intra-abdominal adipose tissue deposition, leading to central obesity. Insulin resistance [27] and enhanced glucocorticoid activity [28] may be responsible for this phenomenon. Also, reduced estrogen exposure [29] occurs during each pregnancy, with multiple pregnancies amplifying the likelihood of MetS and other metabolic disorders. In this regard, a study of the Hispanic/Latina population in the United States showed that those with four births had the highest odds of overall MetS (OR = 1.4, 95%CI 1.0, 2.0) [30]. In addition, in a cohort of 6,157 Chinese women conducted by Xie et al. [2], the number of live-birth pregnancies was correlated with an increased risk of MetS in Chinese women aged 40 years and over, especially in postmenopausal women. As known, Mets increases the risk of non-communicable diseases (NCDs) [31], which may be one of the explanations for increased MM with a rise in parity number. Another possible reason for the increased MM in women with high parity is that women with higher parity are more likely to have an unhealthy diet, less physical activity, less favorable lifestyle (e.g. smoking), and poorer access to health care;; these factors may render them susceptible to chronic diseases [21]. Therefore, polypharmacy becomes more prevalent as chronic diseases emerge. Conversely, polypharmacy itself might increase MM due to the side effects of certain medications.

Other findings of this study indicated that the prevalence of hypertensiontended to increase with a rise in the parity number. Similarly, a cohort study found that in comparison to those with two live births, participants with three and four or more live births were at a 25% and 39% higher risk of hypertension, respectively [7].

Also, the findings of an ongoing cohort study showed that higher parity/live birth numbers might be associated with an increased risk of T2DM in Iranian women [12]. In another cohort study, Gaudet et al. [32] reported an association between multiparity and increased diabetes mellitus-related mortality.

Our results showed that women with a higher parity number had a higher prevalence of abdominal obesity (WC ≥ 88 cm) and elevatedWHtRs.Similarly, Haoling et al. showed a positive association between the number of children and obesity in middle-aged women, even after controlling for many confounders [33].In a case–control study on women of Mexican descent, Martı´nez et al. [19] found that the number of pregnancies was positively associated with increased body size, especially central adiposity. Also, WCwasindependently associated with high parity in the Hispanic population, though there was no association between parity number and WHR.In a cross-sectional analysis of the Tongji-Dongfeng cohort study by Li et al. [21], parity number was positively associated with the risk of obesity, especially abdominal obesity. Moreover, many studies showed positive associations between parity number and weight gain or BMI [18, 20, 34–38].

Some mechanisms explain the association between the number of children and obesity, such as insulin resistance [27], hormonal changes secondary to fewer ovulatory cycles [29], increased glucocorticoid activity [28], and accumulation of fat tissue (especially in the femoral area) during pregnancy. Peripheral insulin resistance caused by pregnancy leading to surplus calorie storage might also play an independent role. When the ability of adipose tissue to store excess energy is restricted because of insulin resistance, the triacylglycerol surplus is deposited at undesirable sites such as visceral adipose tissues [39]. Furthermore, parents with several children may be prone to gain weight as a result of their increased food intake, reduced physical activity, or both [33]. Consequently, obesity can lead to chronic conditions, such as hypertension, cardiovascular disease, T2DM, and cancer [40]. Hence, obesity may also contribute to the higher rate of MM observed in women with elevated parity numbers.

The key limitation of this study is its cross-sectional nature, meaning that we could not establish a cause-and-effect correlation. Therefore, were commend the conduction of large-scale prospective studies to assess for causality in explaining the relationship between parity number and the occurrence of MM and polypharmacy during a woman’s life span.

To our knowledge, this is the first study reporting on the association between parity number, multimorbidity, and polypharmacy, representing the main strength of this work. Moreover, the large sample size of our study denotes another strength.

We conclude that the parity number is associated with MM and polypharmacy in Iranian women enrolled in the Azar Cohort Study. Compared to nulliparous women, women with three or more parties were more likely to have MM, even after adjusting for confounding factors. Also, women with five or more parities were more likely to have polypharmacy than nulliparous women. Further studies exploring the pathways (biological, social, and environmental) underlying these relationships will provide clues for preventing morbidity and premature mortality among susceptible and highly parous women in particular.

## Data Availability

The data that support the findings of this study are available from [Dr. Ata Mahmoudpour, amahmoodpoor@yahoo.com] but restrictions apply to the availability of these data, which were used under license for the current study, and so are not publicly available. Data are however available from the authors( Elnaz Faramarzi) upon reasonable request and with permission of [Dr. Ata Mahmoudpour].
